# Unmasking Compartment Syndrome in an Autistic Adolescent: A Case of Median Nerve Compression Following Distal Radius Open Reduction and Internal Fixation (ORIF)

**DOI:** 10.7759/cureus.96984

**Published:** 2025-11-16

**Authors:** Halimah Khan, Muhammad Khalil

**Affiliations:** 1 Medicine, Royal Lancaster Infirmary, Lancaster, GBR

**Keywords:** autism, compartment syndrome, diagnostic overshadowing, fasciotomy, fracture, neurodiversity, pain assessment, surgery, trauma

## Abstract

Acute compartment syndrome is a rare but limb-threatening complication of trauma and surgery. In neurotypical patients, pain out of proportion to injury is a key diagnostic clue. In autistic and other neurodivergent individuals, altered sensory processing and communication barriers can mask or distort pain reporting, delaying recognition. We present an adolescent male with autism who sustained a comminuted right distal radius fracture in a high-speed motorbike collision. Following open reduction and internal fixation (ORIF), he developed median nerve compression and impending compartment syndrome, requiring urgent fasciotomy and carpal tunnel release. This case highlights diagnostic challenges in neurodivergent trauma patients and the need for tailored, coordinated care pathways.

## Introduction

Acute compartment syndrome is a surgical emergency most often associated with lower limb injuries, but can occur in any fascial compartment following high-energy trauma or surgery [1-3]. It arises when elevated intra-compartmental pressure compromises circulation and tissue viability, with diagnosis heavily reliant on detecting pain disproportionate to the injury-particularly pain on passive stretch-alongside evolving neurovascular changes [1,2]. 

In neurotypical patients, this disproportionate pain is a critical early sign. In patients with autism spectrum disorder (ASD), however, altered pain perception, atypical behavioural responses, communication barriers, and reduced interoceptive awareness can obscure classic symptoms [4-8]. Sensory modulation differences and environmental overload in acute care settings may further mask distress [4,5]. These factors heighten the risk of diagnostic overshadowing, where symptoms are attributed to autism rather than underlying pathology [5]. Over-reliance on subjective pain reports can therefore delay recognition of limb-threatening complications. Objective serial examinations and caregiver input are essential in this population [1-3,4]. 

This report illustrates how these factors influenced the presentation and diagnosis of postoperative acute compartment syndrome in an autistic adolescent following distal radius open reduction and internal fixation (ORIF). 

## Case presentation

Presentation

An adolescent male with known ASD and no other medical history presented following a 50 mph motorbike collision in which he collided with a car and was forcefully dismounted. He was alert with a Glasgow Coma Scale (GCS) score of 15, denied loss of consciousness, and reported no neck or back pain. Neurovascular examination was normal across all limbs. 

**Figure 1:**
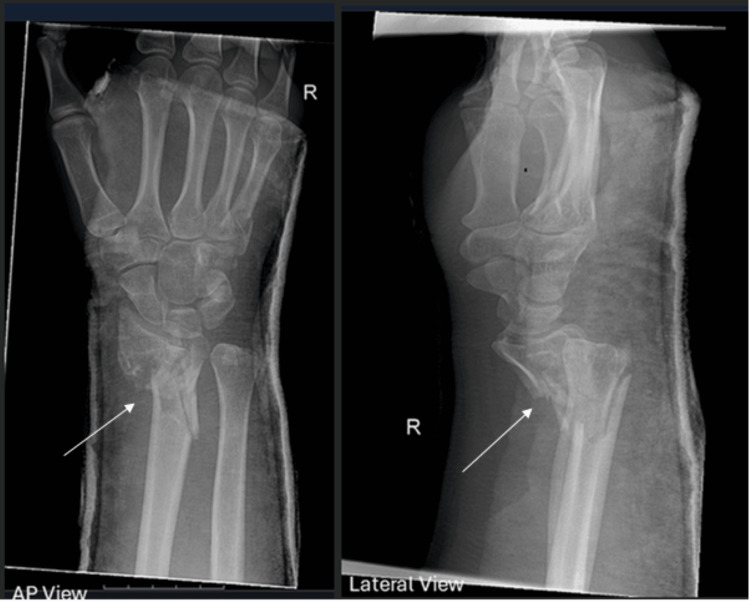
Admission X-rays of the right distal radius showing a comminuted intra-articular fracture with volar displacement (anteroposterior and lateral views). Arrows indicate fracture fragments.

**Figure 2:**
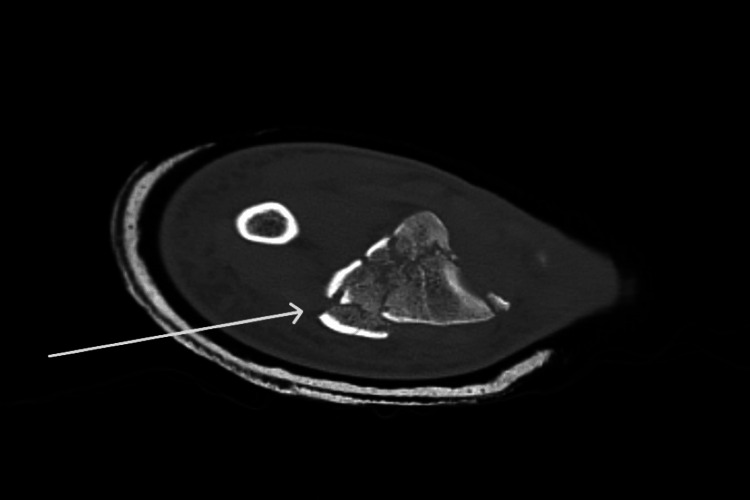
Admission CT X-rays of the right distal radius showing a comminuted intra-articular fracture with volar displacement. Arrows indicate fracture fragments.

Initial investigations, including X-rays and computed tomography (CT) imaging, revealed a comminuted intra-articular fracture of the right distal radius with volar displacement, an undisplaced left acetabular roof fracture, and a left medial tibial plateau fracture (Figures 1, 2). Both lower limb fractures were managed conservatively with strict non-weight-bearing instructions. 

**Figure 3:**
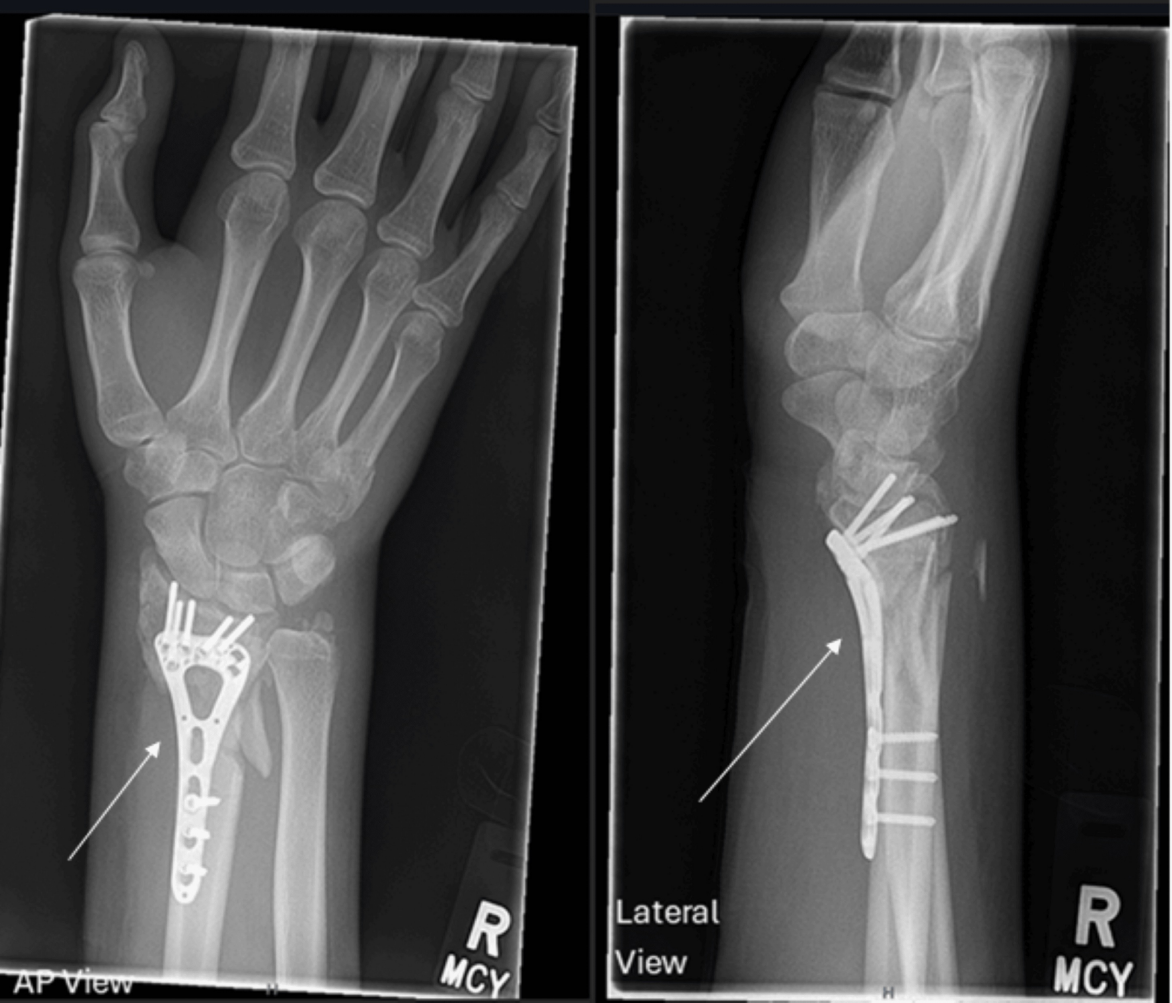
Postoperative X-rays following open reduction and internal fixation (ORIF) of the right distal radius with a volar locking plate (anteroposterior and lateral views). Arrows highlight plate position and fracture alignment.

**Figure 4:**
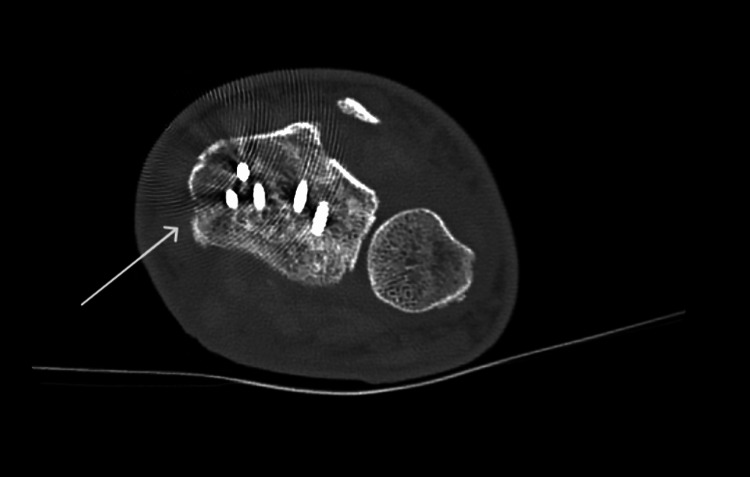
Postoperative CT following open reduction and internal fixation (ORIF) of the right distal radius with a volar locking plate. Arrows highlight plate position and fracture alignment.

The right distal radius fracture required ORIF with a volar locking plate (Figures 3, 4). Intraoperatively, significant forearm swelling was noted. The limb was immobilised in a Bradford sling postoperatively. 

On postoperative day one, the patient reported altered sensation in the right index finger but exhibited minimal behavioural distress, consistent with the atypical pain presentation described in autistic individuals [4-8]. Examination revealed tense forearm compartments and pain on passive stretch, suggestive of impending acute compartment syndrome with median nerve compression [1-3]. 

An urgent re-operation was performed, involving forearm fasciotomy and carpal tunnel release, which led to immediate improvement in sensory symptoms. Postoperative imaging confirmed good plate position and alignment (Figures 3, 4). 

Attempts at primary wound closure were unsuccessful due to persistent swelling. Partial closure was achieved, and the plastic surgery team was consulted for a split-thickness skin graft. Interim care included bi-weekly dressing changes and prophylactic intravenous antibiotics. 

Discharge planning required coordinated input from community nursing, tissue viability specialists, and the Integrated Care Board (ICB). The process was complicated by the patient’s transitional age-too old for paediatric services but without straightforward access to adult community care [9]. 

A summary of clinical events from injury to follow-up is presented in Table 1. 

**Table 1:** Timeline of clinical events from injury to follow-up. ED: emergency department, ORIF: open reduction and internal fixation, ICB: Integrated Care Board, TVN: tissue viability nurse

Date/day	Event(s)
Day 0	50 mph motorbike accident, ED assessment and imaging. Diagnosed. With right distal radius fracture (comminuted, intra-articular, volar displacement), left acetabular roof fracture, and left tibial plateau fracture. ORIF of the right distal radius performed.
Day 1	Altered sensation in the right index finger. Exam; tense forearm compartments, pain on passive stretch. Diagnosed with impending acute compartment syndrome and median nerve compression. Emergency fasciotomy + carpal tunnel release.
Day 2-4	Attempts at wound closure unsuccessful due to swelling. Partial closure achieved.
Day 5	Plastics referral for skin graft.
Discharge	Community wound care arranged via the ICB and TVN.
Follow-up	Ongoing dressing changes. Awaiting definitive grafting.

## Discussion

**Figure 5:**
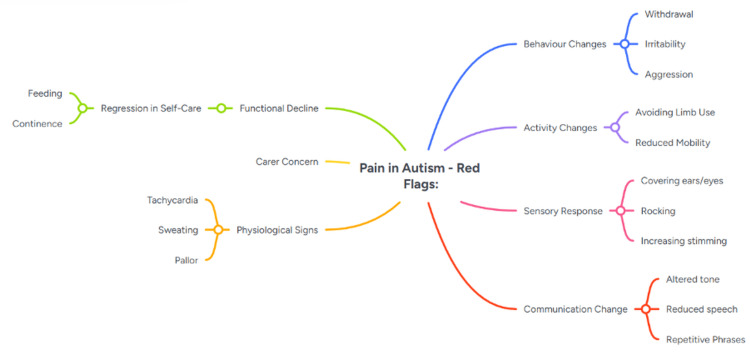
Red flag presentations for pain in autism Illustration of red-flag pain presentations in autism. Diagram summarising atypical behavioural cues for pain compared with typical pain behaviours. Lines outline atypical cues. Original illustration by authors, adapted conceptually from Riquelme I et al. (Neural Plast, 2016) [4] and Bogdanova OV et al. (Front Psychiatry, 2022) [5].

This case demonstrates multiple diagnostic challenges in managing acute compartment syndrome in neurodivergent patients: 

Atypical pain presentation in autism: ASD can involve hypo- or hypersensitivity to pain, altered nociceptive processing, and reduced interoceptive awareness [4-7,10]. Pain behaviours may be replaced by withdrawal, aggression, or sensory avoidance, rather than verbal reporting [4,5,8]. Sensory overload in acute settings may further obscure clinical cues [4,5]. The absence of overt distress in our patient could have been misinterpreted as stability; instead, objective neurovascular findings prompted urgent intervention, avoiding irreversible damage (Figure 5).

Over-reliance on subjective pain reporting risks delayed diagnosis of acute compartment syndrome in this population [1-3,4]. Serial objective examination, caregiver input, and vigilance for behavioural change are critical. Our case avoided diagnostic delay through prompt recognition of examination findings, despite minimal verbal pain reports. 

Collateral information from caregivers often identifies subtle behavioural or functional changes that indicate pain or deterioration [8]. In this case, the mother’s observations supported clinical suspicion and facilitated timely escalation. 

This case also exposed systemic care gaps. Our patient’s discharge planning was hampered by limited integration between paediatric and adult community services, highlighting the need for inclusive pathways for wound care and rehabilitation in this group [9]. 

The decision to proceed to urgent fasciotomy was made on objective signs rather than subjective pain reporting, highlighting the importance of serial examinations and a low threshold for intervention in neurodivergent patients [1-3,4]. 

## Conclusions

In autistic adolescents, compartment syndrome may present without the hallmark pain behaviours typically used to trigger diagnosis. High clinical vigilance, family input, and reliance on objective neurovascular assessment are essential. Coordinated, inclusive care pathways are required to address the transitional gap between paediatric and adult services for complex trauma cases. 

In this case, early recognition and prompt intervention for compartment syndrome prevented long-term neurological damage. However, discharge planning for young people with complex wounds and mobility issues remains an area in need of more inclusive, coordinated systems.
